# Down-regulated and Commonly mutated *ALPK1* in Lung and Colorectal Cancers

**DOI:** 10.1038/srep27350

**Published:** 2016-06-10

**Authors:** Hsien-Feng Liao, Hsien-Hsiung Lee, Ya-Sian Chang, Chia-Li Lin, Ting-Yuan Liu, Yu-Chia Chen, Ju-Chen Yen, Ya-Ting Lee, Chien-Yu Lin, Shih-Hsiung Wu, Ying-Chin Ko, Jan-Gowth Chang

**Affiliations:** 1 The Ph.D. Program for Cancer Biology and Drug Discovery, China Medical University and Academia Sinica, Taichung, Taiwan; 2 Epigenome Research Center, China Medical University Hospital, Taichung, Taiwan; 3 Department of Laboratory Medicine, China Medical University Hospital, Taichung, Taiwan; 4 Institute of Biological Chemistry, Academia Sinica, Taipei 11529, Taiwan; 5 Environment-Omics-Disease Research Centre, China Medical University Hospital, Taichung, Taiwan; 6 Graduate Institute of Clinical Medical Science, China Medical University, Taichung, Taiwan; 7 School of Medicine, China Medical University, Taichung, Taiwan

## Abstract

The *ALPK1* gene located in the 4q25 region encodes a newly explored protein kinase which could phosphorylate the amino acid of a domain full of α-helices. Recently, several studies have indicated that the expression of *ALPK1* is related to inflammation and various diseases; therefore, the purpose of this investigation was to determine whether the expression of *ALPK1* has an influence on tumorigenesis and to further scrutinize its gene polymorphism in order to better understand its clinical importance. In lung and colorectal cancer tissues, the *ALPK1* RNA level of the normal part was higher than that of the tumor part using the RT-qPCR analysis. Moreover, differences in HRM melting curves could effectively separate the known mutation sites and be used to identify the two novel variants that might cause the bio-dysfunctions of ALPK1 found *in silico* predictions. Additionally, in both Lovo colorectal and A549 lung cancer cells with enhanced and depleted expression of *ALPK1*, the encoded *ALPK1* could exert its activity on cell migration without interfering with cell viability. Taken together, these findings suggested that *ALPK1* might play a vital role in cancer development and that the newly explored SNPs are found in a Taiwanese cohort.

In recent years, it has been believed that the progression of cancer from inflammation is activated by inflammatory cells and a variety of mediators, such as cytokines, chemokines and enzymes, which form an inflammatory microenvironment^1^. Relatedly, epidemiological studies have suggested that chronic inflammation may tend to initiate cancer through DNA damage or mutations that are affected by reactive oxygen species and some nitrogen derivatives^2^. According to clinical research, patients with an inflammatory bowel disease, such as Crohn’s disease or ulcerative colitis, have a high risk of suffering from colorectal cancer^3^. In addition, with regard to inflammation of the respiratory system, research has suggested that the more serious and prolonged the inflammatory disease experienced by a patient, the higher his or her risk of developing cancer would be^4^. As such, inflammation has come to be regarded as an enabler of cancer in light of its contributions to core aspects of the disease and the strong evidence of its association with cancer progression in clinical studies, to the extent that it can even be seen as one of the hallmarks of cancer among the eight biological capabilities acquired during the multistep development of the disease^5^.

The encoded α-kinase 1 (*ALPK1*) is a member of the α-kinase family, which, in contrast to other protein kinases, can specifically recognize the alpha-helical conformation part as the phosphorylation site^6^. It is generally acknowledged that ALPK1, which is located in post-*trans*-Golgi vesicles, plays a crucial role in protein selection and space polarization involving myosin Ia in epithelial cells^7^. An inflammatory response can be activated by ALPK1 through the activation of nuclear factor-κb and mitogen-activated protein kinase, a capacity which is proven by the decreased expression of IL-1β and TNF-α in the *ALPK1* knockdown of THP1 cells triggered with monosodium urate monohydrate (MSU)^8^,^9^. In a study investigating the correlation between *ALPK1* and diabetic glomerulosclerosis, it was observed that the ALPK1 found in atrophic renal tubules might contribute to chronic inflammation of the kidneys^10^. Relatedly, atherosclerotic plaques hindering the flow of blood into coronary arterial walls have been shown to cause the transient activation of inflammation, the association of which to myocardial infarction might be attributed to the effect of vascular inflammation^11^. Thus, the aforementioned studies have indicated that the encoded *ALPK1* might have a considerable influence on the development of inflammation in a variety of tissues.

Given the critical importance of identifying target genes linking cancer susceptibility to cancer prognoses, the number of studies aimed at identifying the mutation sites of specific genes has risen sharply in recent years. Thus far, however, there have been few investigations devoted to exploring the correlation between *ALPK1* and cancer development. In light of the considerable evidence demonstrating the relationship between *ALPK1* and inflammation, the present study sought to further clarify the function of *ALPK1* in tumorigenesis, and to identify any gene polymorphisms of *ALPK1* in clinical cases.

## Results

### Detection of *ALPK1* mRNA level in clinical lung and colorectal cancer tissues

**Figure 1:**
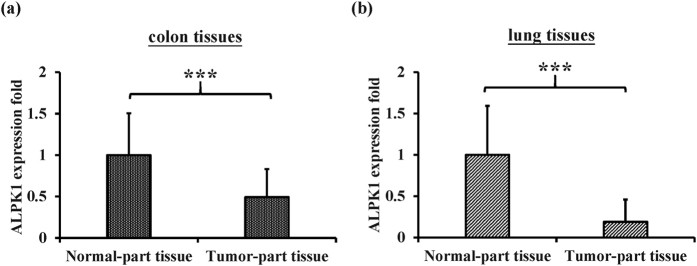
The mRNA expression of *ALPK1* was determined by RT-qPCR in the tumorous and non-tumorous tissues of (**a**) the colorectal cancer and (**b**) the lung cancer patients. ***P < 0.001.

With a view to testing whether *ALPK1* could be involved in the progression of cancer, RT-qPCR (reverse-transcription quantitative polymerase chain reaction) assays were performed to determine the expression of *ALPK1* in lung and colorectal cancer tissues. The results indicated that, in comparison to samples of adjacent normal tissue, both the lung and colorectal cancer tissues exhibited lower mRNA expression of *ALPK1* (Fig. 1a,b). This suggested that lower levels of *ALPK1*, which encodes the main phosphorylation enzyme of myosin Ia known as a tumor suppressor in the intestine^12^, might lead to cancer progression in epithelium-related cancers such as lung and colorectal cancers.

### Screening of *ALPK1* mutations in clinical samples of lung and colorectal cancers by HRM (high resolution melting) analysis

**Figure 2:**
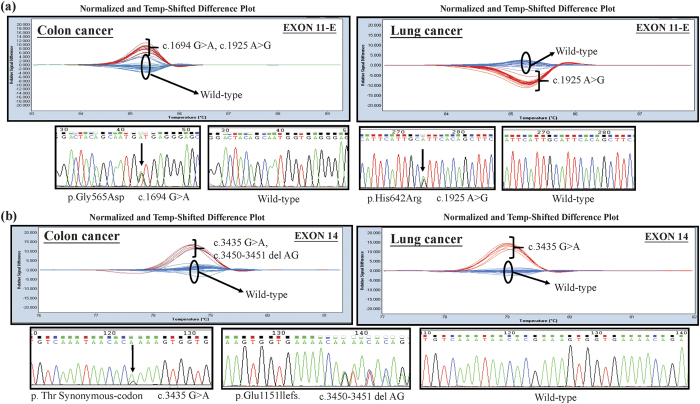
Detection of *ALPK1* sequence variants with (**a**) exon 11-E and (**b**) exon 14 in colorectal cancer and lung cancer patients illustrated by the normalized and temp-shifted difference plot as well as by subsequent confirmation of the example cases using Sanger sequencing, respectively.

**Figure 3:**
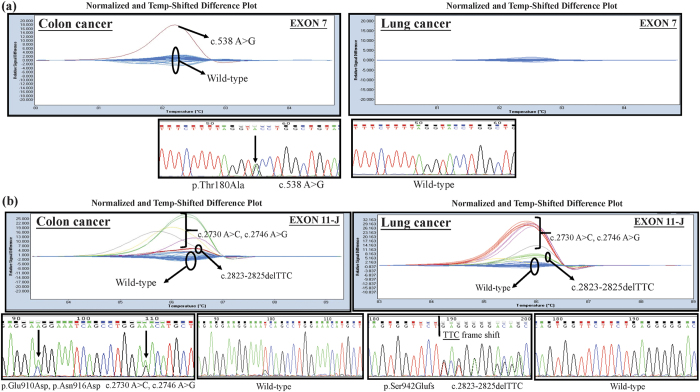
Representative cases of *ALPK1* sequence variants with (**a**) exon 7 and (**b**) exon 11-J in colorectal and lung cancer patients presented by the normalized and temp-shifted difference plot, followed by subsequent confirmation of the example cases using Sanger sequencing, respectively.

According to the collected Cosmic database (Table 1), the ratio of *ALPK1* point mutations accounted for 2.29% of all the mutations in the lung cancer samples and 3.71% of those in the cancer of the large intestine samples. As a result, we further explored the mutation sites of *ALPK1* in the lung and colorectal cancers of a Taiwanese cohort via HRM analysis. Based on the melting profile of *ALPK1* mutations shown in Fig. 2(a,b), the mutations found in exon11-E and exon14 could be clearly and accurately identified in the difference plot curves, and could also be certified by Sanger sequence presented with electropherograms in both lung and colorectal cancers. Among all the mutations that were found, the five known single nucleotide polymorphisms (SNPs), including rs2074388, rs13148353, rs35308602, rs2074381 and rs55840220, and one known frameshift caused by AG deletion in rs201890181 were identified clearly by HRM. Interestingly, the other two unknown mutations containing an A538G resulting in Thr180Ala in exon7 (Fig. 3a) and a c.2823-2825 TCC deletion of exon 11 causing a Ser942Glufs (Fig. 3b) were also found in this determination, and were then further scrutinized to determine the traits of these variants.

### Confirming the novel mutation sites of *ALPK1* using the peripheral blood leukocytes (PBL) of healthy people

**Figure 4:**
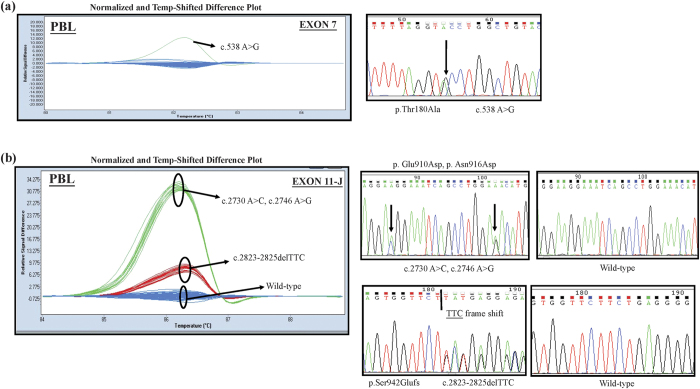
Newly explored variants found in (**a**) exon7 and (**b**) exon11-J certified by testing the PBL of the healthy people in HRM analysis and confirmed by directly sequencing abnormal curves.

To optimize the HRM analysis for novel mutations found in lung and colorectal cancers, control DNA originating from 95 normal PBL templates was used to confirm the novel mutation sites occurring in exon 7 and exon 11-J. Examples of atypical melting curves and the electropherograms of the Sanger sequence identified variation are shown in Fig. 4(a,b). The c.538 A > C in exon 7 was harbored in only one colorectal cancer patient’s tissue, and the melting curve presenting the same mutation site was scrutinized in the normal PBL samples. Moreover, the TTC deletion in exon 11-J was harvested from five lung cancer samples and from four colorectal cancer samples, in addition to also being detected in sixteen normal PBL samples (Table 2). The results implied that the c.538 A > C in exon 7 and the TTC deletion in exon 11-J might be novel SNPs worthy of further exploration with a large-scale population, particularly in terms of their relationship with tumorigenesis.

### Prediction of the structural and functional alterations of the newly found *ALPK1* mutants

**Figure 5:**
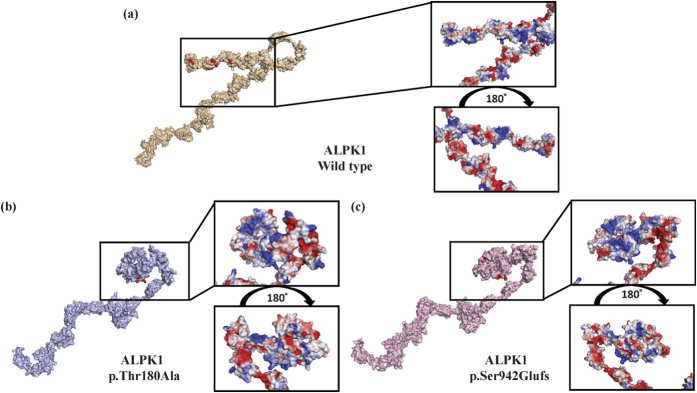
The ATP-binding sites colored in red are shown in three-dimensional virtual models of (**a**) ALPL1 wild type, (**b**) exon 7 mutant and (**c**) exon 11 mutant, and the electron-density distribution is shown with a zoomed-in snapshot of the kinase domain in these three models. (Blue, white and red indicate positive, neutral, and negative potential, respectively).

Modeling of the three-dimensional structures of the wild-type and newly found *ALPK1* mutants revealed obvious differences in terms of their protein shapes and electron-density distributions (Fig. 5a). The ATP-binding activity in these two mutants might be reduced compared to the wild type in that the steric hindrance of the kinase domain restricts the ATPs from attaching to the target sites. Moreover, the alteration of electrostatic surface potential occurring in the kinase domain of the mutant types might affect their substrate-binding affinity (Fig. 5b,c). In the SIFT algorithm, the exon7 mutant-type (p.Thr180Ala), whose score was 0.005 lower than the cutoff, was predicted to be damaging^13^, and the mutation from threonine to alanine at position 180 was found to be unstable based on the lower DDG (The free energy change) value of −1.55 kcal/mol at PH 7 and a temperature >7 °C using the I-MUTANT 2.0 server^14^. In a PROVEAN analysis, the score of the exon 11 frameshift mutant-type (p.Ser942Glufs) was lower at −3.22, which was classified as deleterious^13^. Based on the results of these *in silico* predictions and the virtual structure simulations, it can be concluded that these two newly explored mutations probably cause the bio-dysfunctions of *ALPK1*.

### Exploring the impact of proliferation and migration in the alteration of the encoded *ALPK1* of the Lovo and A549 cancer cells

**Figure 6:**
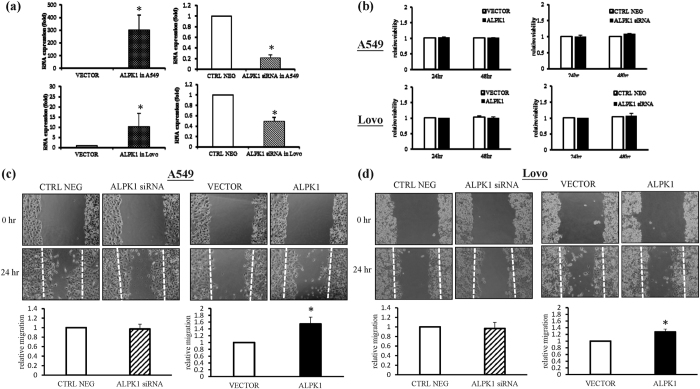
Knockdown and overexpression of *ALPK1* have no influence on cell viability, but do have an influence on cell migration. The Lovo and A549 cancer cells were transfected with siRNA targeting *ALPK1* and *ALPK1* vector for 24 h, which was validated by the RT-qPCR (**a**). Modified cancer cells were tested via MTT viability assay (**b**) and wound healing assay (**c,d**). Data were representative of three independent experiments and *P < 0.05 indicated that there was a significant difference between the control group and the modified cancer cell group. (CTRL NEG: scrambled siRNA sequence; VECTOR: cells transfected with the empty vector; ALPK1: cells transfected with *ALPK1*).

According to previous research, *ALPK1* may influence tumor progression through the phosphorylation of myosin Ia, which is related to the cell orientation polarization and the activity of a tumor suppressor in colorectal cancer^7^,^12^. Lately, however, there have been no studies that have elucidated the function of *ALPK1* in colorectal and lung cancer cells. Thus, we explored whether *ALPK1* leads to tumor progression in Lovo and A549 cancer cells. Firstly, we successfully transfected a siRNA and *ALPK1* vector into Lovo and A549 cancer cells so that the mRNA expression of *ALPK1* could be reduced in the *ALPK1* knockdown cancer cells, while the expression of *ALPK1* would be accelerated in the cancer cells that already over-express it compared to the mock group (Fig. 6a). According to MTT colorimetric assays, the forced expression of *ALPK1* caused no obvious change in the viability of the Lovo and A549 cancer cells after 24 and 48 hours, nor was their viability changed in the *ALPK1* knockdown group (Fig. 6b). Nevertheless, in a wound scratching assay, the ratio of the recovered region was slightly increased in the Lovo and A549 cancer cells transiently transfected with *ALPK1* vector, whereas the area of recovered region was not altered in the *ALPK1* knockdown cancer cells compared to the negative control group within 24 hours (Fig. 6c,d). These results implied that *ALPK1* might play an imperative role in the tumor metastasis of colorectal and lung cancer cells.

### Determining whether the actin distribution would be altered in the Lovo and A549 cancer cells with knockdown and overexpression of *ALPK1*

In accordance with the data presented above, we further hypothesized that the encoded *ALPK1* might cause the alteration of the cell ultrastructure. Subcellular double staining with actin and DAPI was performed in *ALPK1* knockdown and overexpression cancer cells, and then observed using confocal microscopy. The results showed that there were more fusiform cells and actin polymerization in *ALPK1* overexpression A549 cancer cells compared to the other groups (Supplementary Figure S1a). In the Lovo cancer cells, even though the change of the cell shape was not obvious, the average of fluorescence intensity of actin in the ALPK1 overexpression group was more slightly increased than in the other groups (Supplementary Figure S1b). These results suggested that the encoded *ALPK1* might have an influence on cell migration via its effects on the expression of actin.

## Discussion

Colorectal cancer is a disease originating from the epithelial cells lining the colon or rectum of the gastrointestinal tract, and it is characterized by aggressive carcinomas that include a loss of cell polarity, differentiation, and tissue scaffolding, resulting in a poor prognosis among patients with the disease^15^. It was previously reported that myosin Ia possesses the capacity for tumor suppression through its regulation of the polarization and differentiation of colorectal cancers^16^. In previous reports, α-kinase 1 (ALPK1), one of the components of the apical transport machinery, has been identified as an important source for modulating polarized trafficking in enterocytes, and high expression levels of *ALPK1* have been found to promote myosin Ia phosphorylation and regulation in epithelial cell differentiation and polarization^7^. Thus, *ALPK1* might play a key role in the prognosis or diagnosis of colorectal cancer due to its effects on cancer progression.

Given the potential importance of the novel mutation sites in *ALPK1*, such as the Thr180Ala of exon7 and a c.2823-2825 TCC deletion of exon 11 in our Taiwanese cohort, the archived TCGA database (Supplementary Table S1) was adopted for comparison with our findings. Intriguingly, not only were novel variants uncovered in this study; in addition, the known SNPs in *ALPK1*, namely, rs2074388, rs13148353, rs35308602, rs2074381, rs55840220 and rs201890181, were also confirmed in our Taiwanese cohort, even though these SNPs have not yet been included in the TCGA database. Meanwhile, these mutation sites and SNPs were also not found in the Cosmic archives (Supplementary Tables S2 and S3). These interesting phenomena implied that these SNPs might be more highly related to colorectal and lung cancers in the Taiwanese population, in addition to suggesting the new insight that *ALPK1* may modulate colorectal and lung cancer susceptibility or prognosis.

In a simple two-stage carcinogenesis model, the initiation of tumorigenesis is induced by carcinogen-triggered irreversible gene alteration and then promoted by disordered gene expression caused by epigenetic mechanisms and host-selective stressors such as gene polymorphism^5^. Nevertheless, cancer development is actually a multistep process, during which the trait of malignant growth is formed in transformed cells, including insensitivity to antigrowth signals, unregulated proliferation potential, enhanced angiogenesis, metastasis, and so on^16^. The lower expression of *ALPK1* in the tumor tissue samples of cancer patients implied that the disturbance of cell space polarization due to the reduction of *ALPK1* expression might contribute to the initiation of tumorigenesis. In spite of the fact that *ALPK1* slightly enforced the ability of cancer metastasis in the Lovo and A549 lung cancer cells, it was supposed that once the proliferation advantage is obtained, cancer cells may enter a second stage in which their population will expand rapidly; that is, the upregulated expression of *ALPK1* may make a cancer more aggressive in this latter stage.

In summary, we performed a small-scale experiment to verify that the down-regulation of *ALPK1* might promote tumor initiation by interfering with the polarity of the transformed cells even though subsequent high expression of *ALPK1* would play an imperative role in the metastasis phase of tumor progression. In addition, we also further scrutinized the mutation pattern of *ALPK1* in the colorectal cancers of a Taiwanese cohort by using HRM analysis. In spite of the fact that the newly found missense type mutation resulting from c.538 A > C in exon 7 and the serine deletion of a serine-rich domain in exon 11 of *ALPK1* was not specific to colorectal and lung cancers, it would be worthwhile to investigate the relevance between these variants of *ALPK1* and the evolution of the variety of cancers in larger ethnic cohorts.

## Material and Methods

### Sample preparahtion and DNA extraction

The investigation related to this clinical study as well as the collected informed consents abided by the regulations of the ethical committee, and were approved by the Institutional Review Board of China Medical University Hospital (protocol number: CMUH104-REC1-008). The genomic DNA of forty-seven formalin-fixed and paraffin-embedded (FFPE) colon cancer and lung cancer specimens were extracted using the proteinase K and QIAamp^®^ micro DNA extraction kit (QIAGEN, Hilden, Germany) following the manufacturer’s protocol. The total mRNA in the remnants of each sample was subjected to isolation according to the instructions of the High Pure RNA Isolation Kit (Roche Applied Science, Mannheim, Germany). Blood samples from ninety-five healthy subjects were obtained at China Medical University Hospital, and all the samples were processed by DNA extraction with NucleoSpin^®^ Blood Kit (Macherey-Nagel GmbH & Co. KG, Düren, Germany).

### Reverse-transcription for complementary DNA, and real-time quantitative polymerase chain reaction (RT-qPCR)

A high-capacity cDNA reverse transcription kit (Applied Biosystems, Foster City, CA) was utilized to synthesize single-strand cDNA from the total mRNA samples. The *ALPK1* primers included in the Roche Universal Probe Library Assay Design Center software (https://lifescience.roche.com/shop/en/tw/home) were used to execute the RT-qPCR, including forward primer 5′-TGACCACCATTTGCTGTCC-3′ and reverse primer 5′-ACGTGCCACGGATATTCAC-3′. The RT-qPCR reaction was run at 95 °C for 10 min, followed by 40 cycles of 95 °C for 10 s, 60 °C for 30 s, and 72 °C for 10 s in the LightCycler^®^ 480 System (Roche Diagnostics). The results presented are the mRNA expression levels of *ALPK1* normalized to that of *GAPDH* by ΔCt value.

### Design of *ALPK1* exon primers for HRM assay

By using the requirements of the Primer 3 software (http://primer3.ut.ee/), the primers of ALPK1, made up of exon 1 through exon 16, were designed for meeting the requirements of the LightCycler^®^ 480 System Gene Scanning Assay (Roche Diagnostics). The primers were synthesized following the molecular biology quality standard (Protech Technology Enterprise Co., Ltd, Taiwan) and are shown in Supplementary Table S4.

### The high resolution melting (HRM) technique and melting curve analysis

The procedure and performance of HRM analysis have been reported in a previous study^17^. Briefly, the 10 μL final volume was composed of 20 ng of DNA, 0.25 μM of primers, 2.5 mM of MgCl^2^, the LightCycler^®^ 480 High-Resolution Melting Master (Reference 04909631001, Roche Diagnostics) 1× buffer involved with Taq polymerase, nucleotides and the dye ResoLight for performing the PCR reaction. The HRM assays were conducted using the software LightCycler^®^ 480 Gene Scanning Software Version 1.5 built in the LightCycler^®^ 480 Instrument (Roche Diagnostics). The PCR program was composed of the initial denaturation–activation step at 95 °C for 10 min, followed by a 45-cycle program (denaturation step at 95 °C for 15 s, annealing step at 60 °C for 15 s, and elongation step at 72 °C for 15 s with reading of the fluorescence). Performance of the melting program required three steps: denaturation at 95 °C for 1 min, renaturation at 40 °C for 1 min, and melting that consists of a continuous fluorescent reading from 60 to 90 °C at the rate of 25 acquisitions per °C.

There were three steps in the melting curve analysis performed by the Gene Scanning Software. Firstly, normalization of the melting curves was measured on the basis of the initial fluorescence of 100% and the fluorescence remnant of 0% after DNA separation. Secondly, the temperature axis of the normalized melting curves shifted to the point where the entire double-stranded DNA was completely denatured. Finally, the difference plots, which make the differences in the melting profiles more obvious, were formed by the melting curve via calculations completed with Gene Scanning Software. If the shapes of the melting curves were different from each other, then the direct DNA sequencing would be confirmed to avoid false negative results.

### Direct sequencing

Sanger sequencing was performed for all the samples to check the results of the HRM analysis. After HRM analysis, the samples were purified using a DNA Clean/Extract Kit (GeneMark; Atlanta, Georgia, USA) and directly sequenced. The sequence reaction was implemented in a final volume of 10 μL, including 1 μL of the purified PCR product, 2.5 μM of the PCR primers, 2 μL of the ABI PRISM terminator cycle sequencing kit v3.1 (Applied Biosystems) and 2 μL of 5X sequence buffer. The sequencing program consisted of a 25-cycle PCR program (96 °C for 10 s for denaturation; 50 °C for 5 s for annealing; and 60 °C for 4 min for elongation). The sequence detection was performed in the ABI Prism 3130 Genetic Analyzer (Applied Biosystems) according to the standard protocols.

### *ALPK1* transfection and knockdown in the Lovo colorectal and A549 lung cancer cell lines

The Lovo colorectal cancer cells and the A549 lung cancer cells were cultured with F12 and DMEM medium (Gibco, Grand Island, NY, USA), respectively, composed of 100 U/mL penicillin/streptomycin and 10% fetal bovine serum (Gibco) in 5% CO_2_ under 37 °C incubator. Based on the manufacturer’s protocol (Invitrogen, Life Technologies Corporation, CA, USA), the confluent cells were transfected with the scrambled siRNA and human *ALPK1* siRNA whose oligonucleotide sequence was F: 5′-UCCAGUAACAGGCUCAUCAUC UCUC-3′ and R: 5′-CAGAGAUGAUGAGCCUGUUACUGGA-3′. Before performing the knockdown step, the fresh siRNA in the Opti-MEM solution (Invitrogen) was mixed with the Lipofectamine RNAiMAX (Invitrogen) for 5 min, and the total solution was added to the pure DMEM medium without antibiotics. In the course of transfection, the cancer cells were transfected with pCMV 10-His-HA-*ALPK1* and pCMV 10-His-HA, which was kindly supplied by Dr. Ying-Chin Ko^8^, using Lipofetamine™ 2000 transfection reagent (Invitrogen) and Opti-MEM I medium (Invitrogen) following the manufacturer’s protocol. The efficacy of depletion and over-expression was validated by RT-PCR as described above.

### Cell viability and wound healing assays

In the colorimetric proliferation assay, cells (1 × 10^4^ cells/well) were processed by *ALPK1* depletion and over-expression in 96-well plates for 24 and 48 hours and then treated with 10 mg/ml of MTT [3-(4,5-dimethylthiazol-2-yl)-2,5-diphenyltetrazolium bromide] for 2 hours at 37 °C. The formazan crystals in DMSO were detected by an ELISA plate reader at 595 nm. Cell viability was determined by comparing the absorbance value with the control at different times. In addition, cells (1 × 10^5^ cells/well) were seeded on a 12-well plate in complete medium overnight and then scratched using a 200 μL pipet tip to create a crossed gap region followed by photographing with a light microscope at 40-fold magnification. After a 24-h incubation period, the uncovered area (migrated cells-free area) of the gap region in the photographed images was measured using Image J analysis software. The relative uncovered area of the gap region in each group was further calculated according to the value of the same group at the beginning (0 h).

### Modeling of ALPK1 and evaluation of the functional impact of the mutant type

The best-fitting models of the native and mutant *ALPK1* protein structures were optimally built on the I-TASSER server^18^, and functional prediction was analyzed by PROVEAN and I-MUTANT 2.0 web server^13^,^14^. Molecular imaging was created using the PyMOL Molecular Graphics System, Version 1.7.4 (Schrödinger, LLC).

### Immunofluorescence staining and microscopy

The 1 × 10^4^ cells were seeded on the coverslips in 24-well plates and transfected as described above. Then, the cells were fixed with 4% paraformaldehyde for 10 min. The fixed cells were permeabilized by 0.2% Triton X-100 (in PBS), followed using 1% bovine serum albumin (BSA) for blocking. Subsequently, the cells were incubated with beta-actin antibody (1:500; GTX110564; GeneTex, North America) overnight at 4 °C. Thereafter, the cells were washed by 1% BSA 3 times, at 5 min intervals, and further incubated with10 μg/mL of Alexa-Fluro-488 rabbit/FTC-conjugated secondary antibodies (A-11008; Invitrogen, Life Technologies Corporation, CA, USA) for 30 min at 37 °C. For nuclear staining, the cells were treated with 1 μg/μl of 4′,6-diamidino-2-phenylindole (DAPI) for 10 min. The over-stained cells were washed by 1% BSA 3 times, at 5 min intervals. Finally, the stained cells were mixed with ProLong Gold antifade reagent to amplify the fluorescence signal, and images were taken using a Leica TCS SP2 confocal microscope (Leica Microsystems; Germany).

### Statistical analysis

Statistical analysis was calculated by using two-tailed Student’s *T* test in Microsoft Excel 2013 (Microsoft, WA, USA). The results were presented as the mean ± standard error of triplicates of each experiment and were thought to be statistically significant when the *P* value was lower than 0.05.

## Additional Information

**How to cite this article**: Liao, H.-F. *et al*. Down-regulated and Commonly mutated *ALPK1* in Lung and Colorectal Cancers. *Sci. Rep.* **6**, 27350; doi: 10.1038/srep27350 (2016).

## Supplementary Material

Supplementary Information

## Figures and Tables

**Table 1:** Ranking the ratio of point mutations in *ALPK1* by the order of prevalence.

Cancer tissue types	Point Mutations		
	Cases	Tested samples	Mutated samples (%)
Endometrium	20	505	3.96%
Large intestine	29	781	3.71%
Lung	32	1397	2.29%
Liver	13	942	1.38%
Ovary	9	791	1.14%
Breast	8	1210	0.66%
Kidney	2	804	0.25%

The data in this table was collected from the Cosmic database (http://cancer.sanger.ac.uk/cosmic/), which was last updated in July 2015.

**Table 2:** The mutations and SNPs of ALPK1 identified in PBL, colon and lung cancer patients.

*Alpk1*exon	Nucleotide	Type	dbSNP ID number	HRM		
				PBL	Colon cancer	Lung cancer
7	A > G	Missense	Novel	1/95	1/47 (1.0)	0/47 (1.0)
11-e	G > A	Missense	rs2074388		16/47	0/47
11-e	A > G	Missense	rs13148353		16/47	23/47
11-j	A > C	Missense	rs35308602		5/47	10/47
11-j	A > G	Missense	rs2074381		5/47	10/47
11-j	TTC deletion	frameshift	Novel	16/95	4/47 (0.20)	5/47 (0.45)
14	AG deletion	frameshift	rs201890181		1/47	0/47
14	G > A	synonymous	rs55840220		6/47	8/47

The template of *ALPK1* whose accession number was NM 025144 was adopted from the NCBI database (http://www.ncbi.nlm.nih.gov/gene/). In the two novel variants, two-tailed P value shown in quotes was measured by Fisher’s test in colon and lung cancers compared with normal PBL.

## References

[b1] Lu H., Ouyang W. & Huang C. Inflammation, a key event in cancer development. Mol. Cancer Res. 4, 221–233, 10.1158/1541-7786.MCR-05-0261 (2006). 16603636

[b2] Fernandes J. V. . The Role of the Mediators of Inflammation in Cancer Development. Pathol. Oncol. Res. 21, 527–534, 10.1007/s12253-015-9913-z (2015). 25740073

[b3] Itzkowitz S. H. & Yio X. Inflammation and cancer IV. Colorectal cancer in inflammatory bowel disease: the role of inflammation. Am. J. Physiol. Gastrointest. Liver Physiol. 287, G7–17, 10.1152/ajpgi.00079.2004 (2004). 15194558

[b4] Borm P. J. & Driscoll K. Particles, inflammation and respiratory tract carcinogenesis. Toxicol. Lett. 88, 109–113 (1996). 8920724 10.1016/0378-4274(96)03725-3

[b5] Hanahan D. & Weinberg R. A. Hallmarks of cancer: the next generation. Cell 144, 646–674, 10.1016/j.cell.2011.02.013 (2011). 21376230

[b6] Ryazanov A. G., Pavur K. S. & Dorovkov M. V. Alpha-kinases: a new class of protein kinases with a novel catalytic domain. Curr. Biol. 9, R43–45 (1999). 10021370 10.1016/s0960-9822(99)80006-2

[b7] Heine M. . Alpha-kinase 1, a new component in apical protein transport. J. Biol. Chem. 280, 25637–25643, 10.1074/jbc.M502265200 (2005). 15883161

[b8] Wang S. J. . Lymphocyte alpha-kinase is a gout-susceptible gene involved in monosodium urate monohydrate-induced inflammatory responses. J. Mol. Med. 89, 1241–1251, 10.1007/s00109-011-0796-5 (2011). 21822924

[b9] Ko A. M. . ALPK1 genetic regulation and risk in relation to gout. Int J Epidemiol 42, 466–474, 10.1093/ije/dyt028 (2013). 23569188 PMC3695596

[b10] Yamada Y. . Identification of chromosome 3q28 and ALPK1 as susceptibility loci for chronic kidney disease in Japanese individuals by a genome-wide association study. J. Med. Genet. 50, 410–418, 10.1136/jmedgenet-2013-101518 (2013). 23539754

[b11] Fujimaki T., Horibe H., Oguri M., Kato K. & Yamada Y. Association of genetic variants of the alpha-kinase 1 gene with myocardial infarction in community-dwelling individuals. Biomed Rep 2, 127–131, 10.3892/br.2013.190 (2014). 24649083 PMC3917049

[b12] Mazzolini R. . Brush border myosin Ia has tumor suppressor activity in the intestine. Proc. Natl. Acad. Sci. USA 109, 1530–1535, 10.1073/pnas.1108411109 (2012). 22307608 PMC3277176

[b13] Choi Y. & Chan A. P. PROVEAN web server: a tool to predict the functional effect of amino acid substitutions and indels. Bioinformatics 31, 2745–2747, 10.1093/bioinformatics/btv195 (2015). 25851949 PMC4528627

[b14] Capriotti E., Fariselli P. & Casadio R. I-Mutant2.0: predicting stability changes upon mutation from the protein sequence or structure. Nucleic Acids Res. 33, W306–310, 10.1093/nar/gki375 (2005). 15980478 PMC1160136

[b15] Wodarz A. & Nathke I. Cell polarity in development and cancer. Nat. Cell Biol. 9, 1016–1024, 10.1038/ncb433 (2007). 17762893

[b16] Mazzolini R. . Brush border myosin Ia inactivation in gastric but not endometrial tumors. Int. J. Cancer 132, 1790–1799, 10.1002/ijc.27856 (2013). 23002058

[b17] Lin Y. C., Er T. K., Yeh K. T., Hung C. H. & Chang J. G. Rapid Identification of FGFR2 Gene Mutations in Taiwanese Patients with Endometrial Cancer Using High-resolution Melting Analysis. Appl. Immunohistochem. Mol. Morphol. 23, 532–537, 10.1097/PAI.0000000000000114 (2014). 25517871

[b18] Yang J. . The I-TASSER Suite: protein structure and function prediction. Nat. Methods 12, 7–8, 10.1038/nmeth.3213 (2015). 25549265 PMC4428668

