# Supplementary material for: Down-regulated and Commonly mutated ALPK1 in Lung and Colorectal Cancers
Source: Sci Rep. 2016 Jun 10;6:27350. doi: 10.1038/srep27350 (PMC4901299; doi:10.1038/srep27350)
Supplement: Supplementary Information [file srep27350-s1.pdf]

Down-regulated and Commonly mutated *ALPK1* in Lung and Colorectal Cancers

Author names and affiliations

Hsien-Feng Liao[1], Hsien-Hsiung Lee[2,3], Ya-Sian Chang[2,3], Chia-Li Lin[2], Ting-Yuan Liu[2], Yu-Chia Chen[2], Ju-Chen Yen[2], Ya-Ting Lee[2], Chien-Yu Lin[3], Shih-Hsiung Wu[1,4] Ying-Chin Ko[5,6 *], Jan-Gowth Chang[1,2,3,7 *]

[1] *The Ph.D. Program for Cancer Biology and Drug Discovery, China Medical University and Academia Sinica, Taichung, Taiwan*

[2] *Epigenome Research Center, China Medical University Hospital, Taichung, Taiwan*

[3] *Department of Laboratory Medicine, China Medical University Hospital, Taichung, Taiwan*

[4] *Institute of Biological Chemistry, Academia Sinica, Taipei 11529, Taiwan*

[5] *Environment-Omics-Disease Research Centre, China Medical University Hospital, Taichung, Taiwan*

[6] *Graduate Institute of Clinical Medical Science, China Medical University, Taichung, Taiwan*

[7] *School of Medicine, China Medical University, Taichung, Taiwan*

Supplementary Data 1

Variant Classification	Variant Type	dbSNP RS	Exon	ChromChange	AAChange
Missense Mutation	SNP	rs147722416	exon15	c.G3692C	p.R1231P
Silent	SNP	novel	exon5	c.G321T	p.A107A
Missense Mutation	SNP	novel	exon11	c.C2654T	p.A885V
Missense Mutation	SNP	rs6533616	exon6	c.A523G	p.N175D
Silent	SNP	rs17044681	exon11	c.C2349T	p.S783S
Silent	SNP	novel	exon11	c.T1389C	p.H463H
Missense Mutation	SNP	novel	exon3	c.C86T	p.S29L
Missense Mutation	SNP	novel	exon11	c.C2654T	p.A885V
Silent	SNP	novel	exon11	c.C1125T	p.V375V
Missense Mutation	SNP	novel	exon5	c.G449A	p.R150H

Table S1. The current clinical cases of checking mutation sites in *ALPK1*and the collected data from the TCGA database (http://cancergenome.nih.gov/) updated until July. 2015.

Supplementary Data 2

Large intestine

AA Mutation	CDS Mutation	AA Mutation	CDS Mutation
p.V7A	c.20T>C	p.D756N	c.2266G>A
p.S29L	c.86C>T	p.A885V	c.2654C>T
p.K55K	c.165G>A	p.A885V	c.2654C>T
p.F61S	c.182T>C	p.L901I	c.2701C>A
p.A86T	c.256G>A	p.S938fs*14	c.2812delT
p.A107A	c.321G>T	p.S957Y	c.2870C>A
p.A144S	c.430G>T	p.S957Y	c.2870C>A
p.R150H	c.449G>A	p.P962L	c.2885C>T
p.D253N	c.757G>A	p.W991R	c.2971T>C
p.V296M	c.886G>A	p.G1000R	c.2998G>A
p.G304C	c.910G>T	p.I1124M	c.3372A>G
p.V375V	c.1125C>T	p.K1125E	c.3373A>G
p.E438K	c.1312G>A	p.P1132S	c.3394C>T
p.H463H	c.1389T>C	p.K1140*	c.3418A>T
p.C467F	c.1400G>T	p.Y1166N	c.3496T>A
p.E496*	c.1486G>T	p.R1231P	c.3692G>C

Table S2. The majority of mutation sites happened in the colorectal cancer, and the collected data from the Cosmic database (http://cancer.sanger.ac.uk/cosmic/) updated until July. 2015.

Supplementary Data 3

Lung			
**AA Mutation**	**CDS Mutation**	**AA Mutation**	**CDS Mutation**
p.K58K	c.174G>A	p.P787P	c.2361A>T
p.A93A	c.279G>C	p.S832T	c.2494T>A
p.A107A	c.321G>T	p.S832Y	c.2495C>A
p.A109T	c.325G>A	p.C865Y	c.2594G>A
p.R153P	c.458G>C	p.R884M	c.2651G>T
p.M249I	c.747G>C	p.R884M	c.2651G>T
p.L267L	c.801G>T	p.S960L	c.2879C>T
p.A281G	c.842C>G	p.I969I	c.2907C>T
p.T292M	c.875C>T	p.I969M	c.2907C>G
p.C316S	c.947G>C	p.R973H	c.2918G>A
p.C316Y	c.947G>A	p.T1026M	c.3077C>T
p.E319*	c.955G>T	p.K1042*	c.3124A>T
p.L325L	c.975G>A	p.K1043I	c.3128A>T
p.H511Y	c.1531C>T	p.W1051C	c.3153G>T
p.L531L	c.1593C>G	p.Y1161C	c.3482A>G
p.E628D	c.1884G>T	p.M1086I	c.3258G>A
p.Q761Q	c.2283G>A		

Table S3. The majority of mutation sites happened in the lung cancer, and the collected data from the Cosmic database (http://cancer.sanger.ac.uk/cosmic/) updated until July. 2015.

Supplementary Data 4

Alpk1 exon		Primer sequence (5'→3')	Amplicon size, bp
1	**F**	tacctttcaccgaagcaattccta	278
	**R**	agtcaacacttaagacaaatacttcc	
2	**F**	ttgatcttcctgttcccttatccg	267
	**R**	tgtggaatgtttgcctctaaagtg	
3	**F**	tctttccctctcttttgttcacca	268
	**R**	gtgcctcccatcagaacctc	
4	**F**	ttccttacctgaactctgaccttt	238
	**R**	gtctaaatgcctcacttggggata	
5	**F**	tattaatgaaaatgcctcccacgc	315
	**R**	tcaagtagctcgggagggaag	
6	**F**	cagttcctgctgcaatatatcagt	265
	**R**	aaagcttcttcattcatcagtggc	
7	**F**	tgtgctcatgaagacttctgtga	243
	**R**	cacagtgaaaccatgcagcc	
8a	**F**	atactagctgttcctcccctgt	150
	**R**	aagaagcactagacaaaccacct	
8b	**F**	agcagagttaatatgggcctcc	211
	**R**	actaactgtacctggccctatg	
9	**F**	ctttaggccatggatgagagcag	205
	**R**	ctgagaaagacagccactaaccaa	
10	**F**	agcaaaaccatttcatagcctgtt	260
	**R**	gccactgatgagacacaaagc	
12	**F**	ttcaggtgagctggcgtag	305
	**R**	agcaaggttagctcatcttaca	
13	**F**	accacttttgcattttgttgtgtg	333
	**R**	tacagggacaacacacaaggat	
14	**F**	caaaccaagatttacccaagtcca	283
	**R**	aattcccagtttcacatac	
15	**F**	gtgtgtgtgtgtgtgtatgtat	319
	**R**	gagaaccagatatttgcgagcatc	
16	**F**	tggattggtaatgtgacagacctt	263
	**R**	ctgttttccatgttgctttccttg	

(Continued to the next page)

Alpk1 exon		Primer sequence (5'→3')	Amplicon size, bp
11a	**F**	gtcctgattcacttgcagttttca	338
	**R**	tgaaattgtacagcttccccattg	
11b	**F**	tggaaaacaggagcttcacag	292
	**R**	tttatccaacctgcactcgaaact	
11c	**F**	gaagttatgtctgtgattgcccag	324
	**R**	ttccctctgaacattcttacccat	
11d	**F**	cggtgtgtgaagtatttgaaagtg	354
	**R**	aagcactggaagtctggctg	
11e	**F**	cattctgatgcatttcgagtctcc	308
	**R**	tccaaattgtcattgttgggttcc	
11f	**F**	gaaaagagcctggcaaagaacatc	308
	**R**	agaatcagaagaccaagaagcaga	
11g	**F**	cctcataataccccaggcattttc	327
	**R**	taagggtgcatcttcagtgctttc	
11h	**F**	agaagcctttgaaataattgttgagt	303
	**R**	cagttgcccctcctcatcca	
11i	**F**	aattggcctgttcaaaatcctgac	281
	**R**	atgtttccaggctgatttccttcc	
11j	**F**	gctctcatagactgtgcattctga	334
	**R**	tctagtctctgaaacagccaatca	
11k	**F**	cattttccagtggttcttctgagg	278
	**R**	acctttcatgagctttaagtgatct	

Table S4. The Primer sequences of *ALPK1* for HRM analysis.

Supplementary Data 5

(a)

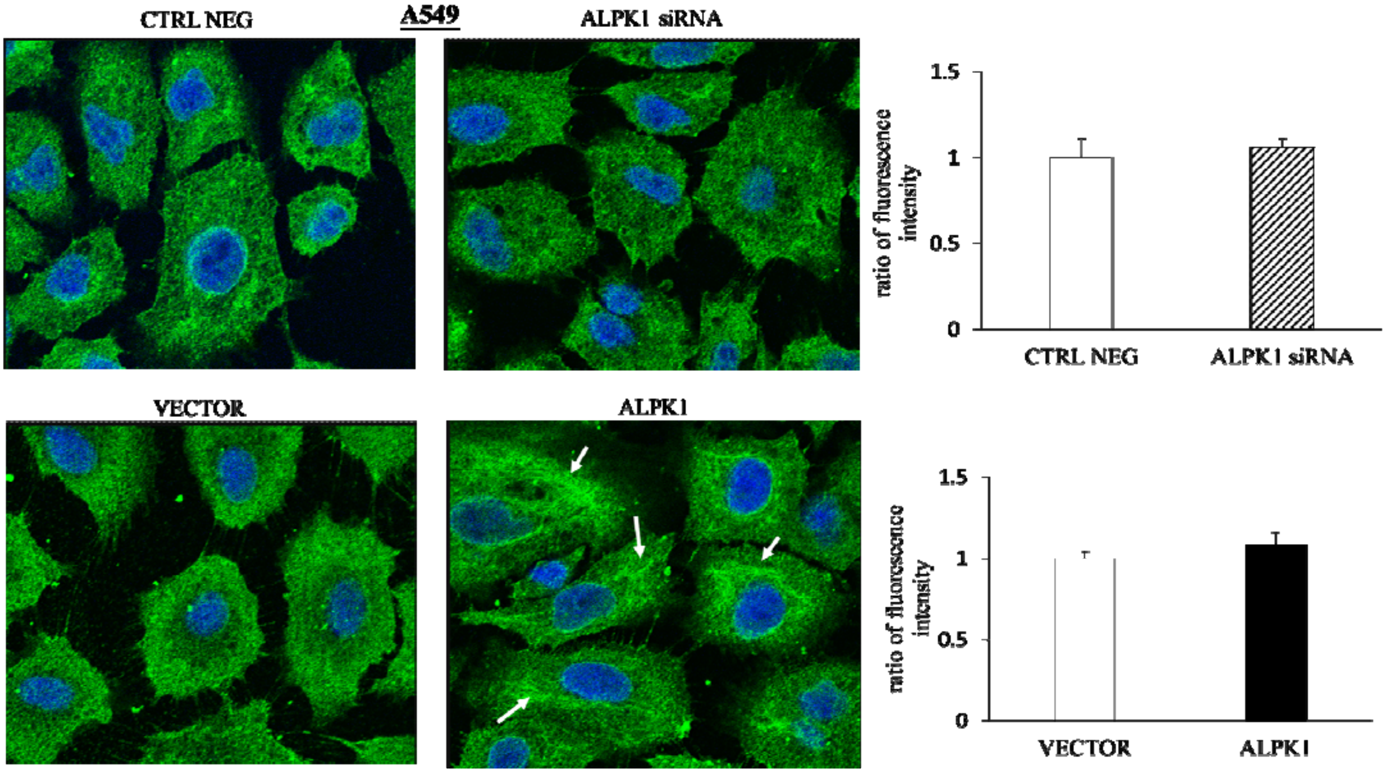

(b)

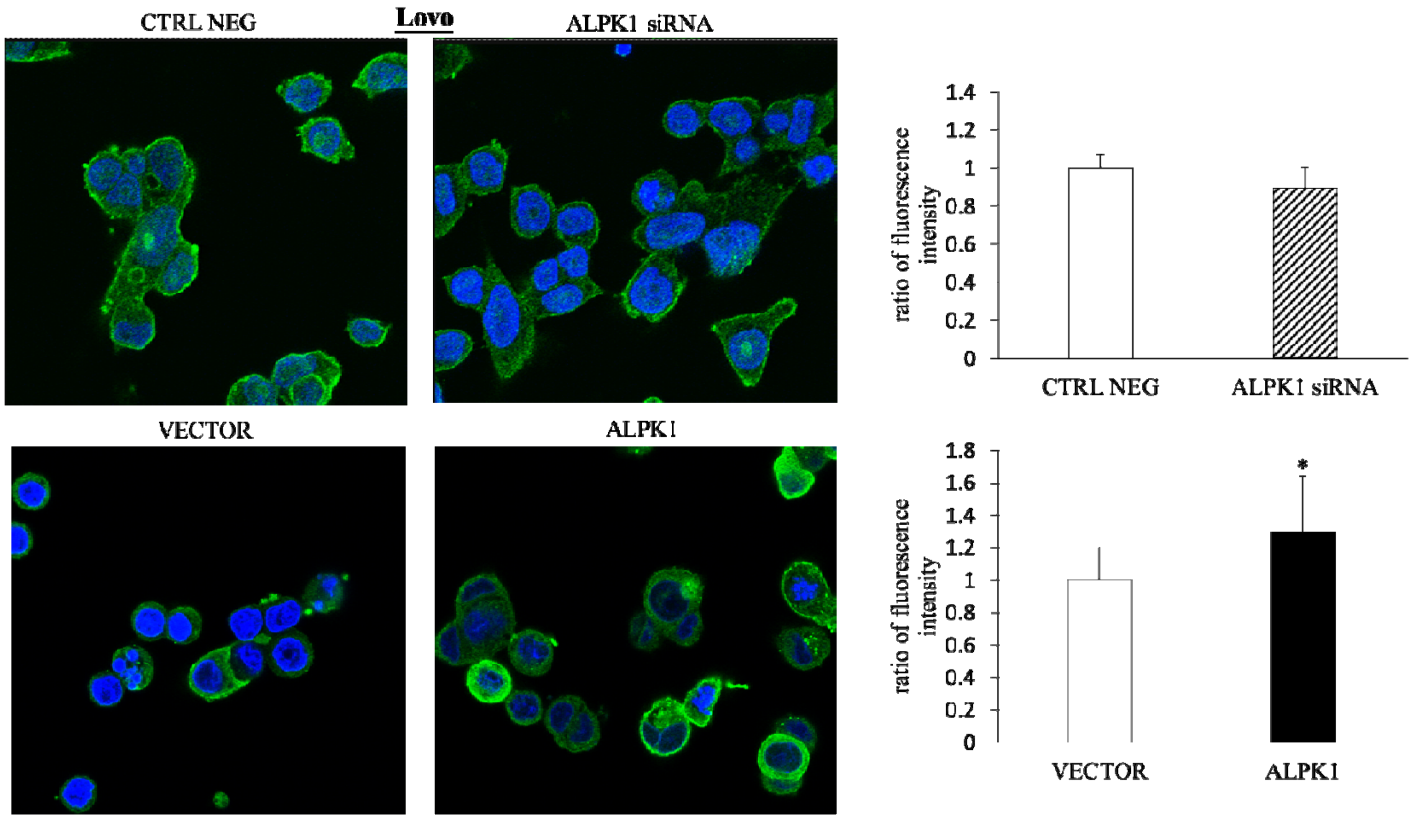

Figure S1.

ALPK1 caused various impacts on the actin distribution. The A549 (a) and Lovo (b) cancer cells were transfected with siRNA targeting ALPK1 and ALPK1 vector for 24h, after which the cells were stained for actin with Alexa Fluor 488 (green) and for the nucleus with DAPI (blue). The white arrows indicate the actin polymerization, and the confocal images (magnification, 1260 X) are shown in the panels on the left. Mean fluorescence intensities of approximately 40 cells per condition in fours random fields were quantified using the ImageJ software program, and data were normalized to the pixel of CTRL NEG or VECTOR, respectively. Quantifications of actin staining are presented in the bar graphs on the right, and * $P < 0.05$ indicates a significant difference

between the two groups.
